# Is *FKBP5 *a genetic marker of affective psychosis? A case control study and analysis of disease related traits

**DOI:** 10.1186/1471-244X-6-52

**Published:** 2006-11-02

**Authors:** Micha Gawlik, Kerstin Moller-Ehrlich, Meinhard Mende, Michael Jovnerovski, Sven Jung, Burkhard Jabs, Michael Knapp, Gerald Stoeber

**Affiliations:** 1 Department of Psychiatry and Psychotherapy, University of Würzburg, Füchsleinstraße 15, 97080 Würzburg, Germany; 2 Institute of Medical Biometry, Informatics and Epidemiology, University of Bonn, Sigmund-Freud-Str. 25, 53105 Bonn, Germany; 3 Department of Forensic Medicine, University of Würzburg, Lindleinstraße 15, 97080 Würzburg, Germany

## Abstract

**Background:**

A dysregulation of the hypothalamic-pituitary-adrenal (HPA) axis has been proposed as an important pathogenic factor in depression. Genetic variants of FKBP5, a protein of the HPA system modulating the glucocorticoid receptor, have been reported to be genetically associated with improved response to medical treatment and an increase of depressive episodes.

**Methods:**

We examined three single nucleotide polymorphisms (SNPs) in *FKBP5*, rs4713916 in the proposed promoter region, rs1360780 in the second intron and rs3800373 in the 3'-untranslated region (3'-UTR), in a case-control study of Caucasian origin (affective psychosis: n = 248; controls: n = 188) for genetic association and association with disease related traits.

**Results:**

Allele and genotype frequencies of rs4713916, rs1360780 and rs3800373 were not significantly different between cases and controls. Two three-locus haplotypes, G-C-T and A-T-G, accounted for 86.2% in controls. Odds ratios were not increased between cases and controls, except the rare haplotype G-C-G (OR 6.81), representing 2.1% of cases and 0.3% of controls. The frequency of rs4713916AG in patients deviated from expected Hardy-Weinberg equilibrium, the genotype AA at rs4713916 in monopolar depression (P = 0.011), and the two-locus haplotype rs1360780T – rs3800373T in the total sample (overall P = 0.045) were nominally associated with longer continuance of disease.

**Conclusion:**

Our data do not support a significant genetic contribution of *FKBP5 *polymorphisms and haplotypes to affective psychosis, and the findings are inconclusive regarding their contribution to disease-related traits.

## Background

Collaterally to a complex dysregulation of neurotransmission, hyperactivity of the hypothalamic-pituitary-adrenal (HPA) axis has been proposed as an essential pathogenic factor in depressive disorders [1]. As a predictor of clinical improvement normalisation of the HPA-system is one of the basic findings [2] as well as a partial cortisol resistance among depressive patients resulting in an impaired negative feedback and elevated cortisol levels [1]. In search for the genetic and functional mechanisms underlying the HPA-dysfunction in depression, recent research focused on genes involved in HPA-axis regulation, such as the genes for corticotropin-releasing hormone or the glucocorticoid receptor (GR) with several cochaperones. Among these *FKBP5 *gained growing interest.

*FKBP5*, synonymous to *FKBP51 *and *FKBP54*, is located on chromosome 6p21, a chromosomal region associated with bipolar disorder and psychosis [3] and consists of 10 exons spanning ~156 kb (UCSC Genome Browser, release August 2004). FKBP5 is mainly expressed in brain, and in a wide range of human cell tissue, including muscle, liver, and thymus. Functionally, cortisol induces the FKBP5 expression by glucocorticoid-response-elements in human lymphocytes [4]. The protein of 457 amino acids is composed of three domains, two immuno-suppressants binding sites and a tetratrico-peptide-repeat domain [5]. FKBP5 reduces the receptor affinity for cortisol by a complex interaction with the mature hetero-oligomeric glucocorticoid receptor complex and is thought to diminish the dynein binding and nuclear translocation of the glucocorticoid-receptor complex [6]. In addition, FKBP5 has been associated with glucocorticoid-resistance in new world monkeys which results from both expression of GRs that are less responsive and overexpression of FKBP5 that further reduces GR responsiveness [7].

In a mutation scan aiming to detect genetic equivalents of HPA-dysregulation among the cochaperon gene cluster in depression, thirty single-nucleotide-polymorphisms (SNPs) were detected at the FKBP5 locus, but no overall genetic association with disease was traceable in a panel of 317 patients with depression [8]. Three SNPs, namely rs4713916 in the putative promoter region, rs1360780 in the second intron, and rs3800373 in the 3'-UTR region, however, were found associated with improved response to antidepressant treatment. Patients with the rs1360780TT genotype had more than twice as many depressive episodes in the past. In lymphocyte cell-culture studies FKBP5 levels were increased for the risk TT-genotype, and the correlation of plasma cortisol and FKBP5 mRNA level was interpreted as a firm control of the HPA-axis by FKBP5 [8]. Given these genetic and clinical findings, we tried to replicate the association of the FKBP5 gene locus with depression and disease-related variables in a case-control study of probands with similar ethnic background.

## Methods

The sample encompassed 248 cases (154 males) with recurrent depression and bipolar disorder according to ICD10 with a mean age of 48.1 years. [9]. Patients also fulfilled the more restricted criteria of monopolar depression (n = 57), and manic depression (n = 191; Table 1) in differentiated psychopathology [10]. Leonhard's conception of manic-depression displays some important differences compared to current conceptions as of bipolar affective psychoses. Manic-depression in differentiated psychopathology represents a distinct clinical and nosological entity. The essential criteria are bipolarity with a melancholic or manic basic syndrome, or presence of mixed states or partial states with lability of the affect in a unipolar course with complete remission after each episode. By contrast monopolar affective psychoses are characterized by distinct affective syndromes recurring in each episode in the same form. In our sample cases with manic-depression encompassed 77% of the total sample of affective psychoses.

**Table 1:** Demographic and disease related traits in the sample of probands with affective psychosis according to differentiated psychopathology

	**Monopolar depression **(n = 57)				**Manic depressive illness **(n = 191)				**Total sample **(n = 248)					
	Male (n = 32)		Female (n = 25)		Male (n = 122)		Female (n = 69)		Male (n = 154)		Female (n = 94)		All cases (n = 248)	

	Mean	SD	Mean	SD	Mean	SD	Mean	SD	Mean	SD	Mean	SD	Mean	SD

Age at onset (years)	42.1	16.5	41.6	15.9	32.3	12.1	32.3	12.9	34.3	14.3	35.1	15.1	34.6	14.5
Age at first hospitalisation (years)	47.9	17.9	45.1	13.3	36.2	13.6	38.8	14.7	38.6	15.3	40.5	14.5	39.3	15.0
Age at assessment (years)	53.8	16.8	55.9	11.8	44.6	15.4	48.7	15.1	46.6	16.1	50.7	14.6	48.1	15.6
Period of time in hospital (sum in weeks)	33.2	24.8	47.8	53.2	38.5	56.6	46.9	58.9	37.4	51.6	47.1	57.2	41.1	53.9
Duration of disease at assessment (years)	12.3	14.2	14.6	8.4	12.9	11.2	16.5	13.4	12.8	11.9	15.0	13.0	14.0	12.4
Suicide attempts (number)	0.4	0.6	0.48	1.0	0.8	1.7	0.8	1.6	0.7	1.5	0.7	1.5	0.7	1.5
In-patient treatment (number)	3.4	2.8	5.8	4.9	4.8	6.9	5.1	4.8	4.5	6.3	5.3	4.8	4.8	5.8

SD: Standard deviation

Diagnosis in differentiated psychopathology was made by repeated personal examinations of experienced psychiatrists (BJ, GS). The probands were recruited from 1996 to 2005 at the Department of Psychiatry and Psychotherapy at of the University of Würzburg. The Department serves the city of Würzburg (130,000 inhabitants) and the surrounding mostly rural area with a mean of 1150 admissions per year and the full spectrum of psychiatric disorders. With the endpoint in 2005, we assessed the clinical data by chart analysis blind to genotyping (Table 1). Age at onset was defined as age at first contact with psychiatric service, duration of disease was measured by the period of time between first hospitalisation and age at recruitment. The number and duration of in-patient treatments was assessed for all hospitalisations for which medical charts were available. The preponderance of males is reflecting the recruitment procedure on wards for acutely admitted males, and does not necessarily point towards gender differences in affective psychoses. The 188 volunteer control subjects (105 males) with a mean age of 30.2 years were recruited from the blood donor centre at the University of Würzburg. All subjects were unrelated and of German Caucasian descent. The Ethics Committee of the University of Würzburg had approved the study, and informed consent was obtained from all subjects.

PCR for allelic discrimination was performed in a final reaction volume of 20 μl containing 20 ng genomic DNA and 10 μl of 2× TaqMan^®^Universal PCR Master Mix (Applied Biosystems) and 1 μl of 20× TaqMan™ SNP genotyping assay including fluorescent tags specific for the wild type allele and the variant allele. Marker amplification was performed in microtiter plates on Biometra thermocycles (Whatman). PCR amplification conditions were according to the manufacturer's recommendation [10 min at 95°C followed by 15 sec at 92°C and 60 sec at 60°C for 40 cycles]. Allelic discrimination with endpoint detection of fluorescence was performed at 60°C on an ABI prism 7000 sequence detection system followed by analysis with an appropriate software package (Applied Biosystems). Allele calling was independently checked by two operators blind to phenotype.

Fisher's exact test and Armitage's trend test were used to compare allelic and genotypic distributions between cases and controls. Haplotype frequencies and global association were calculated with the program FAMHAP [12], which does not specify a confidence interval for Odds ratios. The exact test proposed by Weir [13] was applied for Hardy-Weinberg equilibrium (HWE). For quantitative traits, we performed the Kruskal-Wallis test and analysis of variance (ANOVA) for single marker analysis and for haplotypes by using haplotype trend regression (HTR) [14]. Overall p-values were corrected for multiple testing [12], the statistics on allele and genotype distribution were uncorrected. Analyses were performed independently for the monopolar sample, the sample with manic-depression, and the combined sample.

## Results

Allele and genotype frequencies of rs4713916, rs1360780 and rs3800373 were not significantly different between cases and controls (Table 2). We observed neither gender differences nor differences in the clinical subgroups according to differentiated psychopathology of monopolar affective psychosis and manic-depression (data not shown). For rs4713916, the genotype distribution in cases deviated from HWE (P = 0.048) due to an excess of heterozygous individuals. The standardized linkage disequilibrium (LD) among controls was D' = 0.751 for rs4713916 and rs1360780, D' = 0.750 for rs4713916 and rs3800373, and D' = 0.971 for rs1360780 and rs3800373. A case-control study with 248 cases and 188 controls had a power of 80% to detect (at α = 0.05) an association with a susceptibility allele, under the assumption that the susceptibility allele has a population frequency of 0.3 and the effect of this allele is recessive with a relative risk of 2.8.

**Table 2:** Allele and genotype distribution of polymorphisms spanning the *FKBP5 *locus

	**Controls**						**Cases**							
	Allele		Genotype				Allele			Genotype				

	1 (%)	2 (%)	11	12	22	HWE	1 (%)	2 (%)	P-value^+^	11	12	22	HWE	P-value*

rs4713916 A/G	110 (29.3)	266 (70.7)	18	74	96	0.50	160 (32.3)	336 (67.7)	0.38	19	122	107	0.048	0.12
rs1360780 C/T	262 (69.7)	114 (30.3)	94	74	20	0.35	338 (68.2)	158 (31.8)	0.66	114	110	24	0.73	0.86
rs3800373 T/G	275 (73.1)	101 (26.9)	102	71	15	0.60	344 (69.4)	152 (30.6)	0.23	115	114	19	0.20	0.87

HWE: P-value of Weir's test for Hardy-Weinberg Equilibrium

+: P-value of Fisher's exact test for comparison of allele frequencies between cases and controls

*: P-value of Armitage's trend test for comparison of genotype frequencies between cases and controls

**Figure 1:**
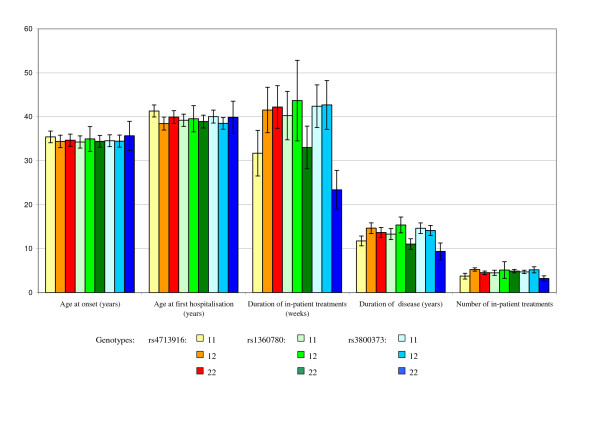
**Genotype-specific mean values of clinical variables in depressive patients for three SNPs in *FKBP5 *(n = 248)**. Using ANOVA and Kruskal-Wallis tests none of the markers reached statistical significance for association with disease-related variables of affective psychosis (I = Mean values including SD).

In the studied population we found two haplotypes, G-C-T and A-T-G, with a cumulated frequency of 86.2% in controls (Table 3). Odds ratios were not increased between cases and controls, except the rare haplotype G-C-G (OR 6.81) representing 2.1% of cases, and 0.3% of controls. Analysis of FKBP5 genotypes and disease related variables produced only one positive association: in monopolar depression (n = 57) rs4713916AA was related to a short duration of disease (P = 0.011; without Bonferroni's correction). Although rs1360780 has been previously described as most significant marker [8], we observed no association of rs1360780 with disease variables (Figure 1), such as age at first hospitalisation (ANOVA P = 0.98; Kruskal-Wallis P = 0.94), number of in-patient treatments (ANOVA P = 0.72; Kruskal-Wallis P = 0.53) or total period in hospital (ANOVA P = 0.67; Kruskal-Wallis P = 0.49). Likewise, the total period of in-patient treatment was not statistically associated with specific genotypes of rs4713916 (ANOVA P = 0.73; Kruskal-Wallis P = 0.82) and rs3800373 (ANOVA P = 0.33; Kruskal-Wallis P = 0.32). We found no association of three-locus haplotypes with clinical variables, neither in the sub-samples nor the combined group (data not shown). Age at onset was neither associated with a distinct genotype nor with a specific haplotype in any of the diagnostic subgroups. However, in the total sample the two-locus haplotype rs1360780T – rs3800373G was associated with longer duration of disease at overall p-value of 0.045.

**Table 3:** Haplotype frequencies of markers at *FKBP5 *calculated by FAMHAP [11]

rs4713916	rs1360780	rs3800373	Controls (%)	Cases (%)	Odds ratio
G	C	T	64.3	60.7	0.86
A	T	G	21.9	24.8	1.18
A	C	T	4.8	5.2	1.10
G	T	G	4.4	3.6	0.80
A	T	T	2.4	2.1	0.88
G	T	T	1.7	1.4	0.82
G	C	G	0.3	2.1	6.81
A	C	G	0.3	0.1	0.51

## Discussion

At the FKBP5 locus allele and genotype frequencies of rs4713916, rs1360780 and rs3800373 were not significantly different between patients with affective psychoses and controls. The putative risk haplotype G-C-G occurred at a frequency of 2.1% among cases with odds ratio of 6.4. Further findings were deviation of HWE among patients at rs4713916, an association of rs4713916 (genotype analysis) and rs1360780 – rs3800373 (two-locus haplotype analysis) with duration of disease. However, we failed to identify a clear-cut association of genetic markers with the clinical course of affective psychosis in the total sample, measured by age at onset, age of first hospitalisation, frequency and period of in-patient treatment.

We confirm the notion of Binder and colleagues [8], that none of the SNPs has a major effect on affective disorder, neither with monopolar depression nor with manic depression. Our sample consisted of German Caucasians identical to the ethnicity of those in the study by Binder et al [8]. The sample comprised 248 patients, 77% of them with a diagnosis of manic depression in differentiated psychopathology [10]. According to this conception, it is possible to diagnose pretended depressive states as episodes of manic-depression if characteristic clinical features are present. Based on these criteria, Pfuhlmann et al. [11] emphasized manic-depression in this strict sense as a nosological entity with an excessive familial morbidity risk of 35.2% among first degree relatives compared to cycloid psychosis (10.8%) and controls (5.7%) in a family study on 431 relatives. In contrast, the sample of Binder et al. [8] consisted of 87% cases with unipolar depression among them an undetermined proportion with bipolar background. It is estimated that at least 10% of unipolar patients switch to a bipolar course in longitudinal studies [15-17].

Regarding the power to detect an association, we had a probability of 80% under the assumption that the susceptibility allele has a population frequency of 0.3 and the effect of this allele is recessive with a relative risk of 2.8 (at α = 0.05). In addition, we observed a two-locus haplotype rs1360780T – rs3800373G associated with a longer duration of disease regardless of the number of sustained episodes (overall P = 0.045) which is in contrast with the observed risk haplotype (rs4713916G – rs1360780C – rs3800373G). The main clinical findings by Binder and colleagues [8] were related to the rare homozygous genotype rs1360780TT, located in intron 2 of FKBP5. The frequency of 9.7% in the present sample was similar to the previous data of 9.1%. Despite this congruence, we found rs1360780TT not associated with an increased frequency of depressive episodes, although our sample had a mean follow-up history of >10 years and a mean number of five episodes requiring in-patient treatment. We assessed these comprehensive clinical data in a retrospective chart analysis, whereas the initial paper [8] mainly analyzed the therapeutic outcome of a single episode in a naturalistic setting over five weeks. Unlike Binder and colleagues [8] we found weak evidence for involvement of rs4713916, located at the presumed promoter region in the vicinity of a putative glucocorticoid response element [4]. Genotype distribution for rs4713916 in patients deviated from HWE and a longer continuance of disease was associated with rs4713916AA in monopolar depression (P = 0.011). Thus, our results may be the effect of chance findings due to sample stratification, although haplotype and haplotype-related analyses were based on permutation-based tests.

Given the differences of LD between the promoter region and distal areas, different genetic variants may be relevant at FKBP5 in depression. Variants at the promoter region may influence the transcription of FKBP5, whereas other functional variants in downstream regions may act as independent risk factors in other cases [18]. Reflecting the complex phenomenology of depression, FKBP5 may play a decisive role only in some cases of depression, and other genes involved in the glucocorticoid system are responsible for the disturbances of the HPA-axis during depressive episodes [19,20]. FKBP5 could be linked to several basic mechanisms of stress related phenomena, as polymorphisms in FKBP5 were associated with peritraumatic dissociation in medically injured children [21].

## Conclusion

In summary, our data do not support a significant genetic contribution of FKBP5 to affective psychosis in the analysed markers, and the findings are inconclusive regarding precisely associated polymorphisms and haplotypes and their association with disease-related traits.

## Competing interests

The author(s) declare that they have no competing interests.

## Authors' contributions

MG carried out the molecular genetic studies and drafting of the manuscript, ME performed laboratory assays, SJ participated in the coordination of the study and MJ performed the chart analysis. BJ participated in the diagnostic evaluation of the patients, MM and MK contributed the data-analysis, interpretation of the data and drafting of the manuscript, GS initiated and coordinated the study. All authors read and approved the final manuscript.

## Pre-publication history

The pre-publication history for this paper can be accessed here:


